# Systematic review and meta-analysis of stress management intervention studies in patients with metabolic syndrome combined with psychological symptoms

**DOI:** 10.1097/MD.0000000000035558

**Published:** 2023-10-20

**Authors:** Ma Lihua, Zhuang Kaipeng, Ma Xiyan, Chang Yaowen, Zhang Tao

**Affiliations:** a The First Hospital of Lanzhou University, Lanzhou, Gansu Province, China; b The 940 Hospital of Chinese People’s Liberation Army, Gansu Province, China.

**Keywords:** intervention, meta-analysis, metabolic syndrome, psychological symptoms, stress management

## Abstract

**Background:**

Metabolic syndrome is affected by many factors, including lifestyle, mood, etc. Self-management of chronic diseases has attracted significant attention from researchers. Some studies have shown that patient self-management is a very important link, which can effectively alleviate the risk of further deterioration of the disease. However, so far, there has been no report on the basis of the summary of self-management intervention programs based on emotion management, which needs further in-depth discussion by researchers.

**Methods:**

The Medline (PubMed), Embase (Elsevier), PsycINFO (Ovid), CBM, CNKI and Wanfang databases were searched from the establishment of the databases to June 2022, and a total of 25 studies were traced. The inclusion criteria on stress management in patients of metabolic syndrome complicated with psychological symptoms uses meta-analysis. Two investigators independently assessed the risk of bias for each study using the Cochrane risk of bias tool.16 studies and 2687participants and relevant characteristics of studies.

**Results:**

In the effects of intervention measures based on stress management on depression, fasting plasma glucose, 2hFPG, high-density cholesterol, self-management behavior and quality of life in patients with psychological symptoms (depression, anxiety, and schizophrenia) complicated with metabolic diseases, there are statistically significant differences between the intervention group and the control group (*P* < .05).

**Conclusions:**

Stress management intervention can effectively improve the health outcomes of patients. In all included analysis indicators, the results of the experimental group are better than those of the control group.

**Key points**

- If the population is in a prolonged state of sustained stress which often causes a series of neuroendocrine responses. The increase of related hormones is closely related to obesity, blood glucose, blood pressure and blood lipid, thus increasing the risk of metabolic syndrome.
- Metabolic syndrome is the pathological state in which multiple metabolic risk factors assemble within the same body, and components mutual correlation, aggregation, is the common soil for chronic diseases, and each other is the cause and effect, forming a vicious cycle, seriously damaging the physical and mental health of patients, reducing the quality of life, leading to heavy economic burden.
- Chronic disease combined with psychological symptoms self-management has become a recognized public health service.

## 1. Introduction

Metabolic syndrome (MS) self-management is an activity in which individuals use the relevant knowledge and skills of health and disease prevention to monitor their own health status and health risk assessment, adjust their own psychology and behavior, improve their health and prevent diseases. At present, the management of stress/emotion in patients with abnormal metabolic indicators of psychological symptoms is still lacking, and further research is urgently needed.

## 2. Methods

### 2.1. Type of research

Randomized controlled trials (RCTs) and quasi-randomized controlled trials were included, and retrospective studies, observational studies, case reports, reviews, and expert reviews were excluded.

### 2.2. Research objects

Considering the formation, impact and intervention mechanism of psychological stress in patients, and according to the methodology of the indirect application of evidence in the Cochrane Handbook for Systematic Review of Interventions,^[1]^ this systematic review ultimately included patients with depression, anxiety or other psychological symptoms combined with MS and abnormal components.

Inclusion criteria: age ≥18 years old; baseline screening of patients with depression, anxiety or other psychological symptoms, development of intervention measures and evaluation of their effects; and members diagnosed with MS, diabetes, hypertension, hyperlipidemia and obesity. Exclusion criteria: pregnant women; and those with other serious physical diseases.

### 2.3. Interventions

The intervention group implemented interventions based on stress management, covering one or more of the following intervention strategies: management plan formulation, including self-management (exercise, lifestyle, medication, and self-monitoring), discharge planning or individualized management; stress/emotion management support, involving cognitive management, behavior management and psychological support management, etc; health education, data provision, and information communication and sharing between providers of stress management interventions and patients (or family members); and other promotion or interventions to improve the current status of patient stress management. Exclusions: intervention strategies that simply target a symptom or behavior of the patient, such as resistance exercise, use of insulin or lipid-lowering drugs, blood glucose monitoring, etc.

The control group received usual care or intervention.

### 2.4. Outcome indicators

Main outcome measures: depression and weight.

Secondary outcome measures: blood pressure, blood glucose and lipid quality of life, protocol compliance, social support, and self-management behavior.

### 2.5. Literature search

#### 2.5.1. Database search.

This research searched the following databases (search platforms): Medline (PubMed), Embase (Elsevier), PsycINFO (Ovid), CBM, CNKI, and Wanfang.

#### 2.5.2. Retrieval strategy.

The search strategy was formulated by combining subject headings and free words, including: stress, mood disorder, depression, post-traumatic stress disorder, anxiety, psychological stress*, psychological disorder*, depress*, and posttrauma*; self-management, stress management, mindfulness-based stress reduction, cognitive therapy, behavioral therapy, cognitive behavior therapy, MBSR, self manag*, stress manag*, mindfulness-based stress reduction, psychosocial treatment, cognitiv*, and behav*; and MS, cardiometabolic syndrome, diabetes, hypertension, hyperlipidemia, and obesity. The languages of the included documents were English and Chinese, and the Chinese documents were limited to Chinese science and technology core journals and above. The time is from the construction of each database to June 2022.

#### 2.5.3. Literature traceability.

This study traced relevant studies that were not identified in the original literature search by examining systematic reviews on relevant topics and references reviewed that met the inclusion criteria.

### 2.6. Information extraction

#### 2.6.1. Screening of included studies.

EndNote X9 software was used to summarize, deduplicate and manage the database search results, and 2 independent investigators performed back-to-back literature screening combined with the inclusion and exclusion criteria.

#### 2.6.2. *Research information extraction*.

Data extraction and management methods followed the standards of the Cochrane Handbook for Systematic Reviews of Interventions. Two researchers extracted the following research characteristics and outcome information from eligible studies: basic information, research objects, intervention content in the experimental group, intervention content in the control group, and outcome indicators.

#### 2.6.3. Research data extraction.

Dichotomous data were extracted in the form of event occurrences and total samples; continuous data were extracted in the form of the means, standard deviations, and total samples. When feasible, the results of intention-to-treat (ITT) analyses were extracted preferentially.

#### 2.6.4. Literature quality evaluation.

Two investigators independently assessed the risk of bias for each study using the Cochrane risk of bias tool.^[2]^

#### 2.6.5. Data analysis.

Data analysis was performed using RevMan 5.3 and Stata 14.0 software. The heterogeneity analysis of the included literature was carried out using the *I*^2^ test. When *P* > .1 or *I*^2^ ≤ 50%, it was considered that there was no heterogeneity, a fixed-effect model was used for analysis, and Begg test combined with a funnel plot was used to judge publication bias. When *P* ≤ .1 or *I*^2^ > 50%, it was considered that there was heterogeneity, the random-effects model was used for analysis, and only Begg test was used to judge publication bias.

## 3. Results

### 3.1. Literature search results

**Figure 1.**
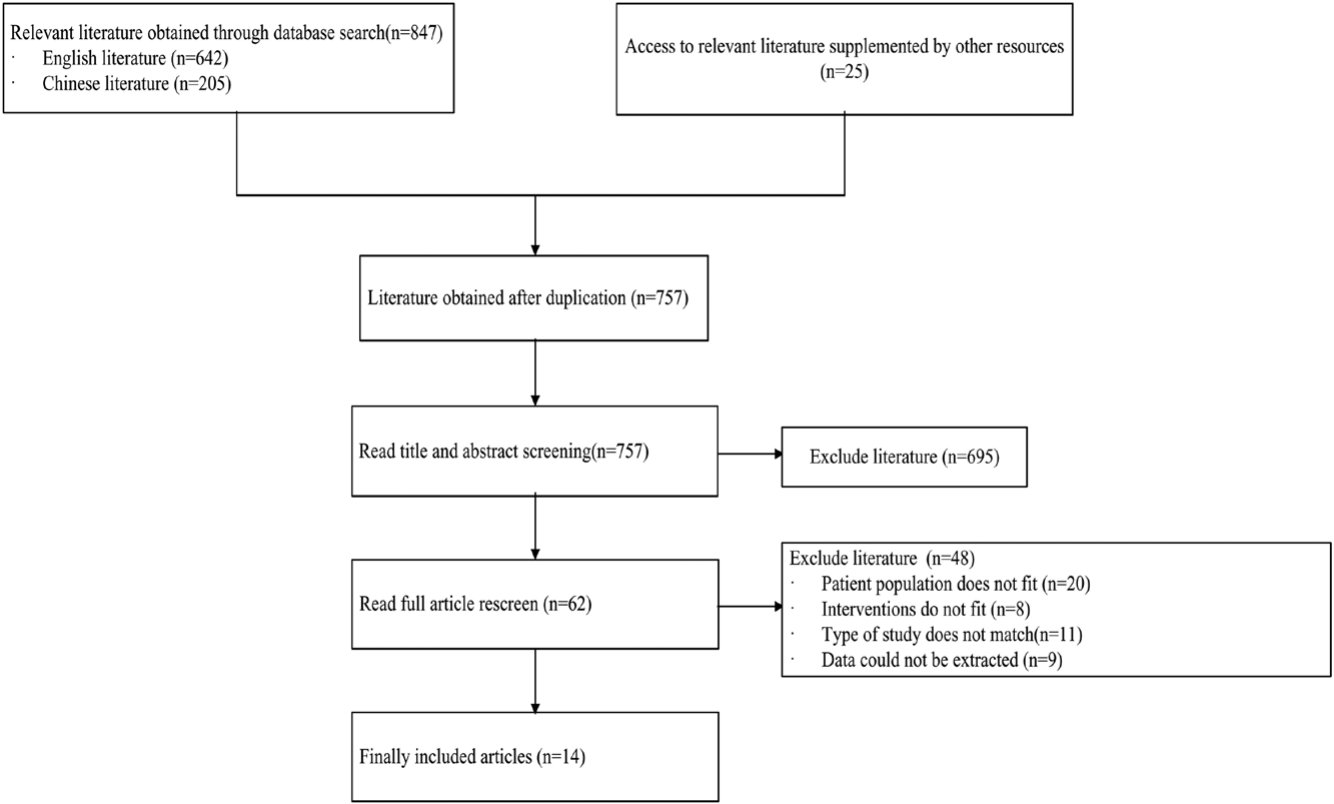
PRISMA flow chart of literature screening.

A total of 847 studies were initially retrieved in this study, including 642 English studies and 205 Chinese studies, and 25 studies were traced through relevant systematic reviews and reviews. The specific search results, screening process, and characteristics of the included and excluded studies are shown in Figure 1.

### 3.2. Basic characteristics of included studies

A total of 14 articles were included in this study, and 14 RCT studies that met the inclusion criteria were extracted. The research areas included China, the United Kingdom, the United States, Croatia, South Korea, and Brazil. The research subjects were all patients with stress, depression or other stress symptoms combined with clinically diagnosed chronic metabolic diseases, mainly diabetes and obesity, although patients with hypertension and hyperlipidemia also were included. The experimental group implemented intervention measures based on stress management, with a variety of interventions, including exercise therapy, individualized health education, self-management, cognitive behavioral therapy, group communication meetings, etc, and most of the studies included intervention measures in 2 or more ways. The intervention time interval was 1 week at most, and the intervention time was generally 60 to 90 minutes. The control group was intervened with at the same intervention frequency and intensity, but the intervention methods were relatively different; they were simple and mainly focused on routine health education and the provision of health education materials. See Table 1 for details.

**Table 1:** Basic characteristics of the studies included in the meta-analysis.

Included studies	Country	Study design	Research object					The intervention content of the experimental group			Content of the control group	Inclusion indicators
			Mental–sympto ms	Diabetes	Obessity	Hyperte-nsion	Others	Type of intervention	Intervention content	Intensity and time		
Williams, 2019^[5]^	UK	RCT	√	√	√	√	√	Relaxation exercise training	1. Teaching: Based on the theory of self-efficacy, it introduces the concept and harm of sedentary life, the benefits of sitting less and moving more, and the relationship between exercise and stress, and it provides information, support and motivation. All participants used a pedometer to record daily activity levels.	1 time every 2 wk, 1 time for 30 min in 16 wk	Conventional treatment. Includes routine care coordination, relevant written information that provides benefits of increased activity.	Depression WC, HDL-C, LDL-C, hypertension
									2. Practical guidance: Partnering with a coach, using a specific movement model that connects the participant head (thinking), heart (feeling), and hands (doing) during the coaching process, enabling participants to understand their own sports situation.			
									3. Outdoor sports: Participants participate in coach-led hikes, mainly walking in local parks. In addition to the benefits of exercise, group participation is emphasized.	1 time a wk, 1 time for 2 h for 16 wk		
Cooney, 2018^[6]^	USA	RCT	√		√		√	Cognitive behavioral therapy (CBT)	1. Provide face-to-face counseling. The counselor worked with the subjects to determine a daily food plan, exercise goals, and stress management goals. Counseling includes weight-loss techniques, emotional management, self-monitoring, social support, and overcoming negative emotions. The subjects recorded daily food intake, physical activity and stress status. 2. Receive a CBT course from a clinical psychologist. Topics include behavioral management and cognitive skills. Course content is highly standardized and follows the brief cognitive therapy manual.	Disease knowledge: 1 time a wk, 1 time for 30 min, for 16 wk	Routine care. Nursing other than cognitive behavioral therapy.	Depression quality of life BMI TG HDL-C
										Psychological consultation: 1 time a wk, 1 time for 30 min, for 8 wk		
Shomaker, 2017^[7]^	USA	RCT	√	√	√			CBT	Conduct group interactive sessions on the interconnectedness of emotions, thoughts, and behaviors, self-monitoring, self-reinforcing, positive self-statements, cognitive restructuring of negative thoughts, and engaging in pleasurable activities. Participants complete weekly homework assignments to apply concepts learned in the course to their daily lives. Small group classes cover nutrition, exercise, conflict resolution, and recognizing signs of depression and suicide.	60 min once a wk for 6 wk	Regular health education.	Depression FPG 2hPG BMI
Jiang, 2016^[8]^	China	RCT	√	√				Cognitive therapy	Explain the mood and blood sugar benefits of maintaining a peaceful mind and exercising appropriately. Explain the impact of improving medication adherence and healthy lifestyle on mood, including rational use of hypoglycemic drugs; Encourage patients to participate in activities within their ability, and convince patients that appropriate behaviors that promote stress relief are beneficial.	1 time a wk for 30 min for 5 wk Follow-up visits were conducted twice a mo; outings were conducted once every 6 mo	General health education model. Includes the predisposing factors of the disease, dietary principles, sleep and medication guidance. After discharge, encourage outpatient follow-up and give guidance and education.	Depression FPG 2hPG quality of life
								Psychologic-al support education	Understand the patient psychological problems and take measures such as counseling, support, comfort, help, and encouragement to guide patients to establish confidence in overcoming the disease. Analyze the causes of negative emotions with the patients and carry out cognitive therapy and supportive education to change the patients cognition of the disease, correct attitude toward treatment and health beliefs. Instruct patients to self-monitor and keep filling out the monitoring diary, including monitoring of mood, blood sugar, blood pressure, weight, and medication, diet management, etc, so that patients can gain a sense of accomplishment during the implementation process and improve their self-confidence.			
Shomaker, 2016^[9]^	USA	RCT	√	√	√			CBT	Interactive session: The first stage includes basic principles and forms of cognitive behavior, affective thoughts and behaviors, mutual introduction, guidance for self-monitoring, cognitive remodeling, and the effect of pleasurable activities on emotions; The second and third stages focus on challenging unsuitable thoughts, making positive self-statements and self-reinforcing; The fourth stage is about positive coping skills for dealing with negative events; The fifth and sixth stages focus on coping with future daily annoyances and life stress. In all classes, participants complete assigned homework, including a mood journal and a record of participating activities, to apply what they have learned in the class to their daily lives.	60 min once a wk for 6 wk	Regular health education.	Depression FPG 2hPG BMI
Xu, 2016^[10]^	China	RCT	√	√				Group sharing session	The group teaching method is adopted, and the content includes reasonable diet, appropriate exercise, emotional management, medication guidance, blood sugar monitoring, etc Specialist nurses act as counselors and use picture-viewing dialogue tools to organize patient discussions and encourage exchanges and experience sharing among patients. The picture dialogue tool includes 4 color pictures, a tutorial guide and several picture dialogue cards. Through the study of color pictures, patients can clearly understand the pathogenesis of diabetes and stress factors and other related knowledge.	2 times a wk, 1 time 60–120 min, for 6 mo	Routine health education.	Depression FPG 2hPG self-management behavior
Pibernik-Okanović, 2015-A^[11]^	Croatia	RCT	√	√				CBT	Interactive group sessions, including thematic presentations and personal experience statements. Topics include recognizing depressive symptoms and dysfunctional thought patterns, coping strategies for reducing depression, understanding bad cognitions, accessing social support, and developing a personal plan for managing emotions/stress.	90 min once a wk for 6 wk	Regular health education. Includes what patients know about themselves, self-control goals, patient concerns, and current test results. Provides written information to address the patient emotional difficulties.	Depression self-management behavior quality of life FPG TG HDL-C
								Emotional self-help handbook	Participants were provided with self-help manuals for overcoming depression, including a manual with practical exercises.			
								Relaxation training (exercise)	Perform warm-up, flexibility, strengthening, and stretching exercises, and motivate patients to increase physical activity in their daily routine. Topics include physical activity for diabetics, the effects of exercise on glycemic control and cardiovascular system and energy expenditure, the effects of exercise on muscles and peripheral nerves and mood, strategies for acquiring physical activity maintenance, and developing a personal plan for regular exercise. Provide official written materials to remind patients to practice and attend meetings.			
Cukor, 2014^[12]^	USA	RCT	√	√		√	√	CBT	When treating patients, one-on-one counseling is provided at the bedside. According to the diagnostic criteria of depression, the content of CBT intervention was modified. Includes the following: psychoeducation, adherence to treatment prescriptions, adaptation of behavioral activation to patient depression, glycemic intervention strategies, and identification of glycemic-specific cognitions to address in restructuring.	1 time a wk, 60 min each time, for 10–12 wk	Routine standard of care. Includes conventional therapy and psychotherapy.	Depression, anxiety quality of life FPG
Bian, 2011^[13]^	China	RCT	√	√				Relaxation training	Inform patients of the importance of exercise and mood and blood sugar regulation. Every afternoon, they go to the rehabilitation center of the hospital for rehabilitation exercises. Patients can jog, climb stairs or play ball games such as badminton and table tennis in the indoor gymnasium. They can participate in agrotherapy work outdoors: vegetable sowing, watering and fertilizing, and picking vegetables. It is advisable to not feel fatigued. Through pulse and blood sugar changes before and after exercise, patients can experience and grasp the physical and emotional state of moderate exercise. After dinner, the nurse arranges the patient to take a free walk in the hospital to eliminate tension and concerns and arouse the patient confidence in healthy self-management.	1 time a wk for 8 wk	General health education. Teaches patients about the etiology, treatment and prevention of diseases in the form of lectures and blackboard reports. In addition, issues a diabetes record booklet, containing health knowledge of diabetes and depression.	Depression anxiety FPG 2hPG
Duarte, 2009^[14]^	Brazil	RCT	√			√	√	CBT	Structural adjustment of the patient treatment plan and introduction to hypertension education, depression and chronic disease symptoms, and cognitive behavior. A cognitive behavioral therapy conference introduces and teaches the main cognitive behavioral therapies: self-monitoring of emotions, cognitive restructuring, planning enjoyable activities, training in social competence and self-confidence, and relaxation exercises with positive imagination. All meetings include corresponding homework assignments.	90 min once a wk for 12 wk, then once a mo for 6 mo	Psychological counseling for the control group. Provides psychotherapy and emotional support. Once a wk, 30–50 min each time, for 12 wk.	Quality of life sleep blood pressure
Fei, 2012^[15]^	China	RCT	√	√				Relaxation training	1. Give patients one-to-one personalized management guidance. Explain to the patient that the role of the educator is to assist the patient to achieve effective self-management. The patient is the main force in the treatment and nursing tasks. The patient is instructed to carry out self-management and self-monitoring according to the contents of the card and participate in a variety of education and relaxation exercises to eliminate tension. Emotions and concerns give patients the hope of healthy self-help. 2. Exercise therapy guidance includes going to the rehabilitation center for whole body exercise every day. Measuring the pulse and blood sugar changes before and after exercise helps patients to experience and grasp the physical and emotional state of moderate exercise. After dinner, the nurse arranges for the patient to take a free walk in the hospital to eliminate tension and facilitate falling asleep. 3. Patients introduce their experiences to each other and describe their participation in education, emotional feelings, and health issues they care about. The mental health status and living habits of the patients are analyzed in detail, and spiritual encouragement is given to those whose symptoms improved.	1 time a wk for 8 wk	Traditional health education. The etiology, treatment and prevention of recurrence of diseases are taught to patients in the form of lectures and blackboard reports.	Depression FPG 2hPG
Lu, 2005^[16]^	China	RCT	√	√				CBT	Guide patients with diabetes-related knowledge to correct patients misunderstandings, eliminate unnecessary pessimism and disappointment, and rebuild their view of health and disease.	2 times a wk, 40–50 min each time, for 4 wk	Usual care treatment.	Depression FPG 2hPG
								Biofeedback training	The patient is active while wearing the EMG biofeedback device, and the doctor gives timely explanation and guidance according to the changes of EMG value using the knowledge of medicine and psychology.	15–20 min 5 times a wk for 4 wk		
Kim, 2011^[17]^	Korea	RCT	√	√	√		√	Psychobehavior-al strategy	Individualized psychobehavioral strategies are provided to participants one-on-one. It consists of 2 stages, and the first stage mainly includes emotional relief, environmental reevaluation, maintenance of interpersonal relationships, current stress status and self-efficacy level. The second stage includes raising awareness, self-assessment, strengthening management and self-efficacy.	Once a wk, 60–90 min for the first time, then 30–40 min each time for 16 wk	Conventional treatment options.	Depression FPG TG blood pressure WC TG HDL-C
								Relaxation training (exercise intervention)	Participants are assessed for preworkout readiness using heart rate detection to obtain exercise intensity load ranges. Determine a participant exercise regimen, with low to moderate intensity, depending on each participant physical condition. Record weekly exercise and food intake. Provides one-on-one telephone counseling to discuss and resolve issues and concerns, including depressive symptoms.	1 time a wk, 150 min each time for 16 wk Follow-up: Once a wk, 10–30 min each time, for 16 wk		
Hu, 2007^[18]^	China	RCT	√	√				Cognitive therapy	In the form of a “diabetes classroom,” demonstrations and videos are used, and the topics include diabetes-related knowledge and patient exchanges. In addition, knowledge manuals and picture albums are distributed, emphasizing the relationship between blood sugar and psychological stress.	Once a wk, 120 min each time for 4 wk. After that, 2 times a mo for 3 mo	Routine hypoglycemia, lipid-lowering drug therapy.	Depression FPG 2hPG self-management behavior
								Supportive therapy	Health care workers care for, support, encourage and comfort patients; mutual patient care, family and social support.			
								Coping style guide	Instruct patients to eliminate stressors, change cognitive assessments, seek social support, and adopt a “transferred” coping style.	Once every 2 mo, 30–40 min each time		
								Behavioral therapy	With the help of the doctor, formulate a 1-wk diet and exercise plan for the patient. It is then recorded truthfully so that the implementation of the plan can be checked.			

2hPG = 2-hour postprandial blood glucose, BMI = body mass index, FPG = fasting plasma glucose, HDL-C = high-density cholesterol, RCTs = randomized controlled trials, TG = triglyceride, WC = waist circumference.

### 3.3. Meta-analysis results

#### 3.3.1. Depression.

**Figure 2.**
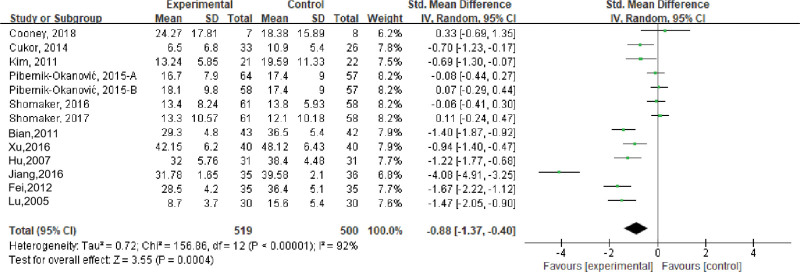
Effects of stress management-based interventions on depression in patients. CI = confidence interval. SD = standard deviation.

Thirteen studies evaluated the effects of stress management-based interventions on depression in patients with psychological symptoms (depression, anxiety, and schizophrenia) and chronic diseases, including 1019 participants. Meta-analysis showed that there was high heterogeneity among studies (*I*^2^ = 91%, *P* < .001), and a random-effects model was used. The results showed that the depression score of the intervention group was lower than that of the control group, and the difference was statistically significant (SMD = −0.88, 95% confidence interval [CI] [−1.37 to −0.40], *P* < .001); see Figure 2.

#### 3.3.2. *Fasting blood sugar*.

**Figure 3.**
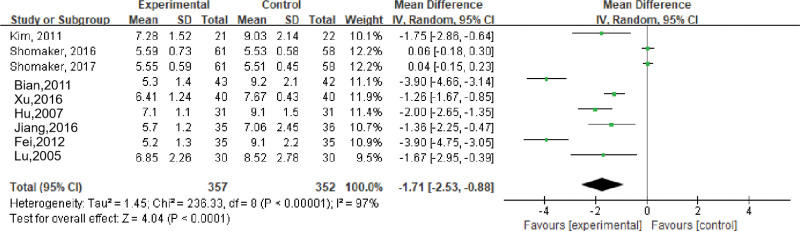
Effects of stress management-based interventions on patient FPG. CI = confidence interval. SD = standard deviation.

Nine studies evaluated the effects of stress management-based interventions on fasting blood glucose in patients with psychological symptoms (depression, anxiety, and schizophrenia) and metabolic diseases, including a total of 709 subjects. Meta-analysis showed that there was high heterogeneity among studies (*I*^2^ = 97%, *P* < .001), and a random-effects model was used. The results showed that the fasting blood glucose of the intervention group was lower than that of the control group, and the difference was statistically significant (MD = −1.71, 95% CI [−2.53 to −0.88], *P* < .001); see Figure 3.

#### 3.3.3. 2-hour postprandial blood sugar.

**Figure 4.**
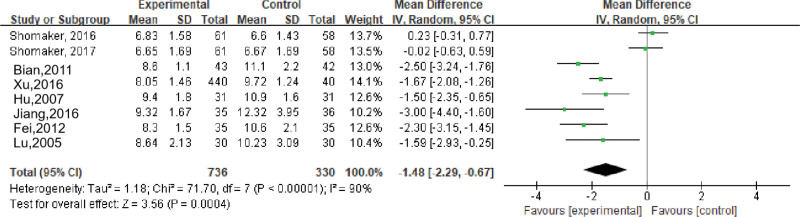
Effects of stress management-based interventions on 2-hour postprandial blood glucose (2hPG) in patients. CI = confidence interval. SD = standard deviation.

Eight studies evaluated the effects of stress management-based interventions on 2-hour postprandial blood glucose (2hPG) in patients with psychological symptoms (depression, anxiety, and schizophrenia) and metabolic diseases, including a total of 1066 subjects. Meta-analysis showed that there was a high degree of heterogeneity (*I*^2^ = 90%, *P* < .001), and a random-effects model was used. The results showed that the 2hPG in the intervention group was lower than that in the control group, and the difference was statistically significant (MD = −1.48, 95% CI [−2.29 to −0.67], *P* < .001); see Figure 4.

#### 3.3.4. *Weight control*.

**Figure 5.**
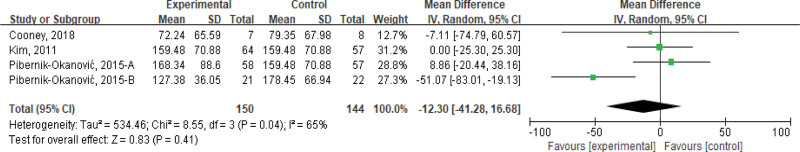
Effects of stress management-based interventions on patient weight. CI = confidence interval. SD = standard deviation.

Four studies evaluated the effect of stress management-based interventions on the efficacy of weight control program management in patients with psychological symptoms (depression, anxiety, and schizophrenia) and metabolic diseases, including a total of 210 subjects. Meta-analysis results showed that there was no heterogeneity among the studies (*I*^2^ = 0%, *P* > .05), and a fixed-effect model was used. The results showed that the weight of the intervention group was lower than that of the control group, and the difference was statistically significant (SMD = −0.34, 95% CI [−0.61 to −0.07], *P* < .05); see Figure 5.

#### 3.3.5. Triglyceride (TG).

**Figure 6.**
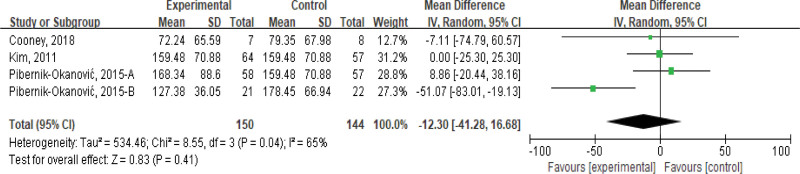
Effects of stress management-based interventions on triglycerides in patients. CI = confidence interval. SD = standard deviation.

Four studies evaluated the effects of stress management-based interventions on TGs in patients with psychiatric symptoms and metabolic diseases, including a total of 294 subjects. Meta-analysis results showed that there was a high degree of heterogeneity (*I*^2^ = 65%, *P* < .05), and a random-effects model was used. The results showed that there was no significant difference in TGs between the intervention group and the control group (MD = −12.30, 95% CI [−83.01, 16.68], *P* > .05); see Figure 6.

#### 3.3.6. High-density cholesterol (HDL-C).

**Figure 7.**
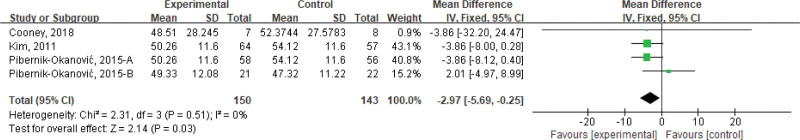
Effects of stress management-based interventions on high-density lipoprotein cholesterol in patients. CI = confidence interval. SD = standard deviation.

Four studies evaluated the effect of stress management-based interventions on HDL-C in patients with psychiatric symptoms and metabolic disease, including a total of 293 subjects. Meta-analysis results showed that there was no heterogeneity among the studies (*I*^2^ = 0%, *P* > .05), and a fixed-effect model was used. The results showed that the HDL-C in the intervention group was lower than that in the control group, and the difference was statistically significant (SMD = −2.97, 95% CI [−5.69, −0.25], *P* < .05); see Figure 7.

#### 3.3.7. Self-management behavior.

**Figure 8.**
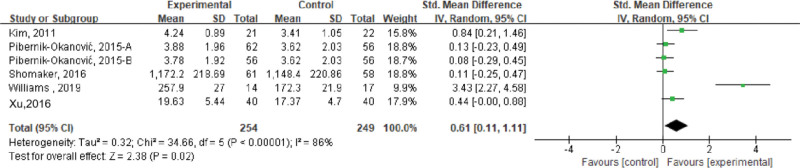
Effects of stress management-based interventions on patient self-management behaviors. CI = confidence interval. SD = standard deviation.

Six studies evaluated the effects of stress management-based interventions on self-management behaviors in patients with psychological symptoms (depression, anxiety, and schizophrenia) and metabolic diseases, including a total of 503 subjects. The results of the meta-analysis showed that there was a high degree of heterogeneity (*I*^2^ = 86%, *P* < .001), and a random-effects model was used. The results showed that the self-management behavior of the intervention group was higher than that of the control group, and the difference was statistically significant (SMD = 0.61, 95% CI [0.11–1.11], *P* < .05); see Figure 8.

#### 3.3.8. Quality of life.

**Figure 9.**
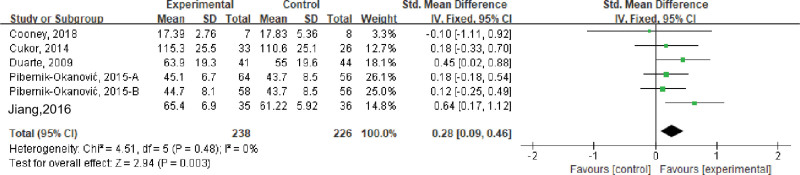
Effects of stress management-based interventions on patients quality of life. CI = confidence interval. SD = standard deviation.

Six studies evaluated the impact of stress management-based interventions on the quality of life of patients with psychological symptoms (depression, anxiety, and schizophrenia) and metabolic diseases, including a total of 464 subjects. Meta-analysis results showed that there was no heterogeneity among the studies (*I*^2^ = 0%, *P* > .05), and a fixed-effect model was used. The results showed that the quality of life of the intervention group was higher than that of the control group, and the difference was statistically significant (SMD = 0.64, 95% CI [0.17–1.12], *P* < .01); see Figure 9.

#### 3.3.9. Risk of bias assessment each primary outcome.

**Figure 10.**
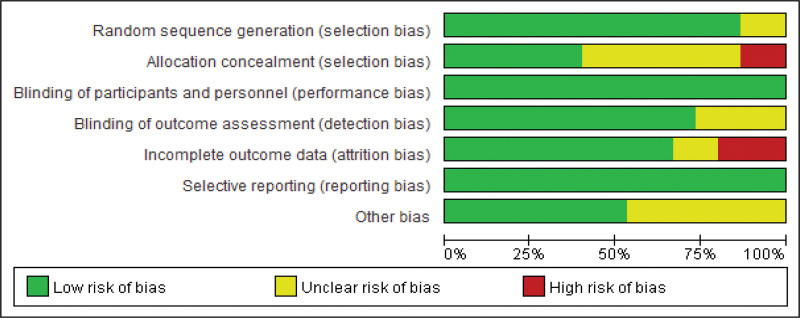
Overall risk of bias assessment results for included studies.

**Figure 11.**
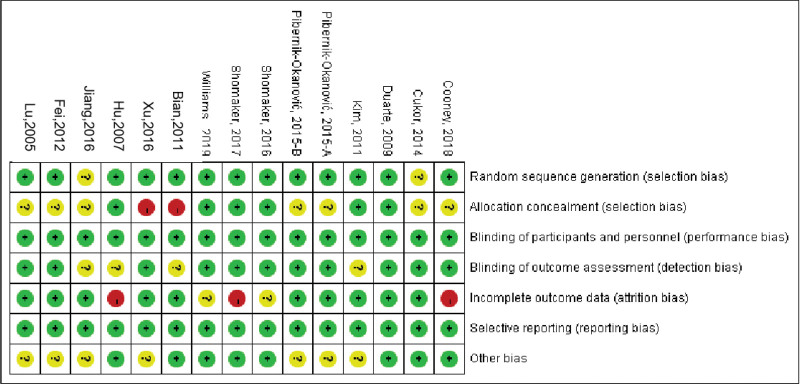
Risk of bias assessment results of the included studies.

The 14 included studies were all RCT studies. The results showed that the quality of the studies was generally high. The high-risk indicators were allocation concealment (2 articles, 14.29%) and incomplete outcome indicators (3 articles, 21.43%). See Figures 10 and 11 for details.

#### 3.3.10. Publication bias.

Publication bias for the primary outcome measures, that is, depression, fasting blood glucose, 2hPG, weight control, TGs, and HDL-C, was calculated using Begg test. The results showed that all the main outcome indicators were *P* > .05, indicating that there was no obvious publication bias in the study, as shown in Table 2.

**Table 2:** Publication bias of included studies.

Metabolic indicators	Adj. Kendall score (P–Q)	Std. Dev. of score	Number of studies	Z (continuity corrected)	Pr > z (continuity corrected)
Depression	−30	16.39	13	1.77	0.077
FGP	−6	9.59	9	0.52	0.602
2hGP	−2	8.08	8	0.12	0.902
Weight control	4	2.94	4	1.02	0.308
TG	−2	2.94	4	0.34	0.734
HDL-C	4	2.94	4	1.02	0.308

2hPG = 2-hour postprandial blood glucose, FPG = fasting plasma glucose, HDL-C = high-density cholesterol, TG = triglyceride.

## 4. Discussion

This systematic review investigated whether stress management-based interventions improved outcomes in patients with psychological symptoms and metabolic disease. The results of the meta-analysis showed that compared with conventional interventions, the patients who implemented the stress/stress management program decreased depression by 0.88, fasting blood glucose by 1.71, 2hPG by 1.48, and weight by 0.31; in addition, quality of life increased by 0.64 and self-management behavior by 0.61. Stress management measures can effectively improve patient health outcomes, and the results of the experimental group were better than those of the control group in all included analysis indicators. Among them, depression, blood sugar, and self-management behaviors had high heterogeneity. In fact, the sources of heterogeneity in the research on stress management-based interventions are relatively obvious. On the one hand, although this article defines the patient depression and other emotional symptoms, the inclusion of metabolic diseases is relatively loose, and the patient disease characteristics are relatively complex. On the other hand, the studies included in this paper applied a variety of stress management methods, and some studies also adopted a variety of integrated intervention measures, which also increased the risk of heterogeneity to a certain extent.

Analysis of the intervention content of the included studies showed that the included studies involved exercise relaxation training, individualized health education, self-management, cognitive behavioral therapy, group communication meetings, etc, and the types of interventions were diverse. Among them, behavioral cognitive therapy and exercise relaxation training are intervention methods with high application rates, and these are also the more mainstream stress management strategies. Cognitive behavior therapy is a general term for a class of psychotherapy methods, including cognitive technology and behavioral cognition.^[3]^ Its interventions include misperceptions, eliminating unnecessary pessimism, reshaping health concepts, and techniques for dealing with negative emotions. In addition, patients are required to complete homework assigned in class, record mood logs and participate in activity experiences to apply what they have learned to their daily lives. Exercise relaxation training, judging from the effects of stress management in the studies included in this paper, can indeed effectively improve the patient emotional management level and the patient metabolic index status. It has been reported in the literature that consistent and regular exercise is integral to maintaining good health,^[4]^ with benefits including improved sleep, which can help promote mental health; release of the body natural “happy” substances—endorphins; access to an exercise and health sense of achievement after status improvement; the elimination of unhealthy thinking patterns; increased blood flow to the brain; improved executive ability; and the promotion of social activities with other bodybuilders. Health education is a traditional nursing intervention measure and an important means for nursing staff to intervene and manage patients. Different from traditional health education, the personalized health education included in this study emphasizes the participation of patients and their families. Medical staff and patients work together to determine daily food plans, exercise goals, and emotional intervention plans and then use them in the subsequent intervention processes according to the individual patients, including situation-tracking adjustments. The duration until the follow-up in the included studies varied from 6 to 16 weeks, the frequency of interventions varied from once a week to once every 2 months, and each lecture was 30 to 90 minutes. To ensure the comparability of the research, the control group was basically intervened with the same frequency and intensity of intervention, but the intervention method was relatively simple.

## 5. Limitations

The heterogeneity of the results of the research indicators is high. The effect size may be imprecise. We did not use controlled vocabulary terms such as MeSH and Emtree in the search strategy. We did not register the review, Due to the limited number of included studies, subgroup analysis could not be carried out according to the different follow-up lengths of the study according to the different stages of disease progression of the patients. These factors affected the results of the study to a certain extent.

## Author contributions

**Data curation:** Chang Yaowen.

**Formal analysis:** Ma Xiyan.

**Funding acquisition:** Lihua Ma.

**Methodology:** Zhuang Kaipeng.

**Resources:** Ma Xiyan.

**Software:** Zhang Tao.

**Writing – original draft:** Lihua Ma.

**Writing – review & editing:** Chang Yaowen, Zhang Tao.
